# Fish Assemblages Associated with Natural and Anthropogenically-Modified Habitats in a Marine Embayment: Comparison of Baited Videos and Opera-House Traps

**DOI:** 10.1371/journal.pone.0059959

**Published:** 2013-03-21

**Authors:** Corey B. Wakefield, Paul D. Lewis, Teresa B. Coutts, David V. Fairclough, Timothy J. Langlois

**Affiliations:** 1 Western Australian Fisheries and Marine Research Laboratories, Department of Fisheries, Government of Western Australia, Perth, Western Australia, Australia; 2 School of Plant Biology, The University of Western Australia, Perth, Western Australia, Australia; University of Canterbury, New Zealand

## Abstract

Marine embayments and estuaries play an important role in the ecology and life history of many fish species. Cockburn Sound is one of a relative paucity of marine embayments on the west coast of Australia. Its sheltered waters and close proximity to a capital city have resulted in anthropogenic intrusion and extensive seascape modification. This study aimed to compare the sampling efficiencies of baited videos and fish traps in determining the relative abundance and diversity of temperate demersal fish species associated with naturally occurring (seagrass, limestone outcrops and soft sediment) and modified (rockwall and dredge channel) habitats in Cockburn Sound. Baited videos sampled a greater range of species in higher total and mean abundances than fish traps. This larger amount of data collected by baited videos allowed for greater discrimination of fish assemblages between habitats. The markedly higher diversity and abundances of fish associated with seagrass and limestone outcrops, and the fact that these habitats are very limited within Cockburn Sound, suggests they play an important role in the fish ecology of this embayment. Fish assemblages associated with modified habitats comprised a subset of species in lower abundances when compared to natural habitats with similar physical characteristics. This suggests modified habitats may not have provided the necessary resource requirements (e.g. shelter and/or diet) for some species, resulting in alterations to the natural trophic structure and interspecific interactions. Baited videos provided a more efficient and non-extractive method for comparing fish assemblages and habitat associations of smaller bodied species and juveniles in a turbid environment.

## Introduction

Marine embayments and estuaries provide important habitats during the life histories of many fish species [1], [2], [3]. The evolutionary importance of these areas is evident through their role in facilitating genetic subdivision and divergence in marine and estuarine fish populations [4], for species that exhibit both high or low dispersal capabilities [5], [6]. However, on a much shorter time scale, anthropogenic activities have modified their seascape through the addition of infrastructure (e.g. piers and rockwalls), removal of substrate (e.g. dredging and mining), or chemical (e.g. eutrophication) and biological (e.g. introduced species) contamination. Despite extensive examples of anthropogenic induced impacts [7], the ecological processes governing sheltered nearshore areas continue to come under stress from further coastal development. Given the ecological importance of embayments and estuaries it is important to understand how such impacts influence their faunal assemblages.

The capacity of anthropogenically-modified (hereafter referred to as modified habitats) seascapes to resemble natural habitats has been evaluated through comparisons of their faunal assemblages [8]. Modified habitats have typically been shown to support a subset of fish species that occur in adjacent natural habitats with similar physical attributes (e.g. topography), and in relatively higher or lower abundances depending on species-specific requirements for shelter, reproduction and diet [9]. Any significant change in the composition of fishes from that of a natural state should be considered an impact based on alterations to the trophic structure and other interspecific interactions which are likely to occur [9]. Results of studies comparing species richness, abundance and composition between natural and modified habitats (ranging from artificial reefs to piers) are inconsistent and have been shown to be influenced by the materials used in their construction and the sampling method [8]. Large habitat modifications could lead to significant changes in the ecological and/or physical (e.g. hydrodynamics) processes within these embayments and estuaries (e.g. influence spawning and recruitment processes, [10]).

**Figure 1:**
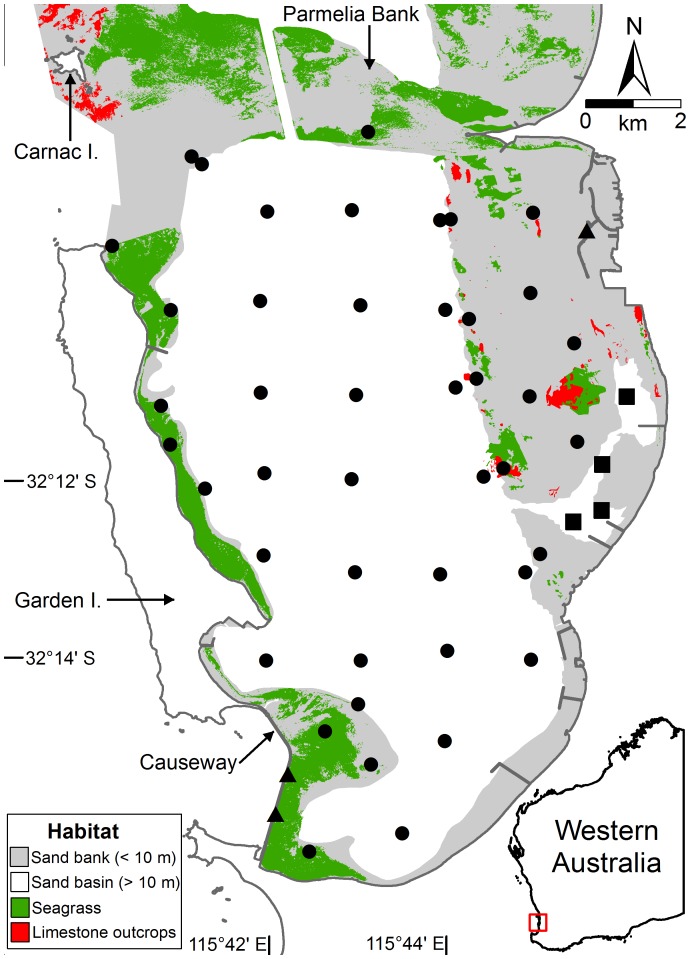
Sites sampled (n = 51) in sand, seagrass and limestone outcrop habitats (black circles) and in dredged channels (black squares) and along rockwalls (black triangles) in Cockburn Sound (habitat map provided by Oceanica Consulting Pty Ltd).

Marine embayments are rare on the west coast of Australia. The temperate embayment of Cockburn Sound (32°12′ S, Fig. 1) is recognised as an important spawning and nursery area for many commercially and recreationally targeted fish and crab species [3], [10], [11], [12], [13]. Cockburn Sound is located in close proximity (ca 20 km) to the only capital city along this extensive coastline (ca 3000 km). As such, this embayment supports competing human uses, including heavy industry, shipping, a naval base, aquaculture and commercial and recreational fishing, with further large-scale port developments proposed. Large-scale declines in seagrass cover (ca 80% loss) have been linked to development activities in this embayment [14], [15], [16]. The few studies of the fish communities of Cockburn Sound have focussed on seagrass beds using beach seine nets, trawling and set (gill) nets [17], [18], [19], [20] and soft sediments using trawling [21], [22]. However, there have been no comparisons of the fish faunal composition using the same sampling method among the different natural and modified habitats, as not all the above methods are able to sample these various habitats effectively.

Baited video offers a non-extractive and effective method for describing and comparing fish communities across multiple habitats [23], [24]. However, this method has had limited application within marine embayments and estuaries [25]. Technological improvements have led to baited video equipment becoming more affordable and once acquired, the method is relatively inexpensive, can be easily repeated and can sample complex topography and sensitive habitats, such as seagrass [26]. While traps are an extractive method, they have been used to effectively sample the fish communities and populations in various habitats [27], [28], [29], [30], [31]. Comparisons between sampling efficiencies of baited video and commercial fish traps from tropical regions have demonstrated that baited video sampled higher numbers of species and abundances, and thus provided greater statistical power for detecting differences in the structure of fish communities [27]. The majority of fish species that occur in Cockburn Sound are either smaller bodied or are typically juveniles [18], [22], compared with those sampled in the previous comparison by Harvey et al. [27].

Given Cockburn Sound is an important recruitment area for *Pagrus auratus* and *Sillaginodes punctatus* [10], [32], our objective was to investigate whether baited videos or traps were the more effective method for assessing small-bodied fish assemblages to determine their composition, relative abundances and associations with four natural (seagrass, limestone outcrops and soft sediment at<and >10 m depth) and two modified habitats (rockwall and dredged channels). As such, the ability of each method to discriminate fish species compositions, species richness and relative abundances at identical locations were investigated. The capacity of the modified habitats to function as natural habitats was assessed by comparing fish species compositions, species richness and relative abundances of fishes recorded in each habitat. If the two modified habitats were adequate substitutes for natural habitats in Cockburn Sound, it was hypothesised that based on the similarities of their physical characteristics, fish assemblages associated with rockwalls would resemble those of limestone outcrops, whereas assemblages in dredged channels would resemble those of soft sediment habitats.

## Materials and Methods

### Study Area and Sampling Regime

Cockburn Sound is a semi-enclosed marine embayment ca 16 km long by 9 km wide and has a sea surface area of ca 100 km^2^ and maximum depth of 23 m (Fig. 1). This embayment is bounded by the mainland to the east and south, Garden Island to the west and the shallow (<10 m) Parmelia Bank to the north (Fig. 1). The southern entrance of the sound has been partially closed through the construction of a rock-filled causeway in 1971–73. All margins of the sound have shallow banks (<10 m) comprising seagrass [33], small outcrops of limestone and extensive soft sediment (Fig. 1). Shipping channels have been dredged into the eastern and northern banks. A dominant feature of the benthos of Cockburn Sound is the deeper central basin (ca 20 m), which is a relatively uniform expanse of soft sediment (silt, Fig. 1). There is extensive industrial development and associated structures along the eastern margin of the sound and boat harbours at the south-eastern end of Garden Island (naval base), either side of the southern end of the causeway and in the north-east corner of the sound (Fig. 1).

Sampling was undertaken in June and July 2008. Baited videos were deployed over four days at 51 sites, followed one week later by traps at the same sites also over four days. Traps were intentionally used after baited videos, as the extraction of fish using traps could potentially reduce abundances and thus influence the results from the baited videos. Sampling sites were determined from ArcGIS^©^ habitat maps (provided by Oceanica Consulting Pty Ltd) and targeted the major natural habitat types including sand banks (<10 m deep, eight sites), central sand basin (>10 m deep, 23 sites), seagrass (six sites) and limestone outcrops (seven sites, Fig. 1). Modified habitats including rockwalls (three sites) and dredged channels (four sites) were also sampled. Three replicate baited videos and traps were sampled concurrently 100–150 m apart at each site. The bait was refreshed for each baited video and trap set with ca 150 g of diced Australian pilchards (*Sardinops sagax*). Baited videos were left to record for 35 minutes, based on the effective duration determined by Morrison and Carbines [34]. Traps were left to soak for 90 minutes as Ferrell and Sumpton [35], using the same type of traps, found catch rates of teleosts reached an asymptote after this period.

### Baited Video and Trap Construction

**Figure 2:**
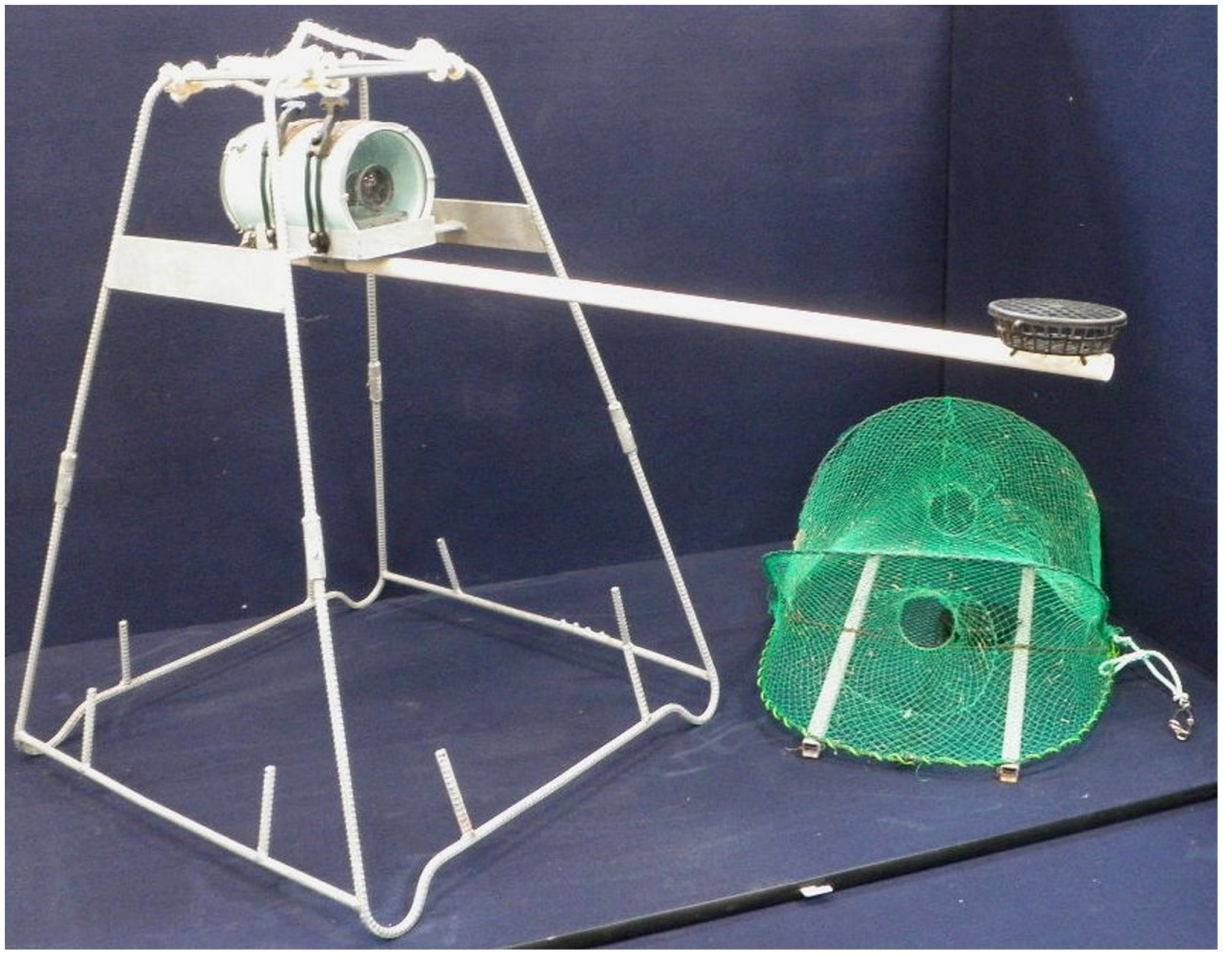
Baited underwater video (left) and opera-house trap (right). Scale reference: camera to bait holder 100 cm, floor to camera 75 cm and trap height 35 cm.

The baited videos consisted of a single high definition (1920×1080 pixels) video camera (Canon HV20) placed in an underwater housing and fastened to a bar situated 75 cm from the floor and centrally within a galvanised-steel trapezium frame with the video orientated horizontally (Fig. 2). Bait was placed in a circular (13.5 cm diameter, 4 cm height) black plastic meshed container and suspended within the field of view 100 cm in front of the camera (Fig. 2). A rope and float for retrieval of the baited video was attached via a rope bridle at the top of the trapezium frame.

A pilot study was conducted to determine 1) an optimal length for the bait pole for baited videos to allow sufficient monitoring around the bait container in the turbid conditions, and 2) the most effective construction for fish traps. The different configurations of fish traps trialled included green vs black 25 mm stretched mesh over both a rounded opera-house trap (90 cm long, 60 cm wide and 35 cm high) and a rectangular trap (93 cm long, 57 cm wide and 33 cm high). The green meshed opera-house trap sampled a greater range of species at higher relative abundances, and was thus used for the remainder of the study. Each opera-house trap had a 10 cm diameter PVC ring spliced into each of the two openings that were located at either end. Two 25 mm square steel channels were attached to the base of each trap to provide ballast and rigidity, thereby minimising potential motion induced from water surge/currents (Fig. 2). Bait holders, identical to those used for the baited videos, were secured within the traps but offset from the two openings (Fig. 2). The traps were retrieved using a rope attached to a float on the waters surface, with the rope attached to the trap at the base midway along one side and perpendicular to the two openings.

### Data Collection and Analysis

Footage recorded from baited video were analysed using a custom interface (BRUVS version 2.1, developed by the Australian Institute of Marine Science) to incorporate data collected from the field, the timing of events, reference images of the seafloor and fish in the field of view. The habitat classifications determined from GIS maps were confirmed for each replicate from video images. Natural habitats were classified into four categories, i.e. shallow sand bank (<10 m, SP), deep sand basin (>10 m, SB), seagrass (GR) and limestone outcrops (LM), and modified habitats into two categories, i.e. rockwall (RW) and dredged channel (DG). Fish were identified to the lowest possible taxa. The relative abundance of each species was determined as the maximum number visible in the field of view at any one time (*N_max_*) for baited video and the number caught for traps. The mean relative abundance of all demersal fish and the total number of species recorded were compared for each habitat and method separately.

The multivariate analyses were performed in PRIMER with the PERMANOVA add-on (version 6.1.13, [36], [37]). The abundance measure of fish species at each site was calculated as the mean relative abundance (*N_max_*, baited videos; numbers, traps) for the three replicates. Considering the more efficient sampling method was to be used for future monitoring following the completion of this study, analyses of each data set were performed separately and thus relative abundances were not standardised between methods. Relative abundance data for both methods were fourth root transformed prior to analyses based on the gradient of the lineal relationship between the logarithms of standard deviation and mean abundances of species, to down-weight the contribution of the most dominant species [38]. Given their tendency to emphasise species composition and relative abundance within community data, zero-adjusted Bray-Curtis and both modified Gower *Log_10_* and *Log_2_* resemblance measures were considered prior to analyses [39]. The stress performance derived from Shepard Diagrams, that displayed the departure of pairwise distances from the best-fitting increasing regression line produced from non-metric multi-dimensional scaling (nMDS) ordination, indicated that baited video and trap data were better treated using a zero-adjusted Bray-Curtis similarity matrix [40].

Assemblage analysis was performed using permutational multivariate analysis of variance (PERMANOVA, [41]). An unconstrained ordination using principal coordinates analysis (PCO) combined with cluster analysis were used to determine fish assemblage groupings among sites. The significance of these groupings were assessed using a similarity profile test (SIMPROF, [36]). These groupings were compared to a constrained ordination using canonical analysis of principal coordinates (CAP), which maintained *a priori* habitat classifications. An appropriate subset of axis (*m*) for the CAP analysis was determined by maximising the leave-one-out allocation success (*m* = 5). The first squared canonical correlation (δ^2^) and leave-one-out allocation success were used as an indication of how well groups were discriminated within the CAP analysis, as they provide a useful statistical estimate of misclassification error and demonstrate how distinct groups of sites are in multivariate space [42]. A Spearman correlation >0.35 was used as an arbitrary limit to display potential correlations between individual species abundances and habitats relative to the canonical axes.

When significant *P* values were obtained from pairwise tests using PERMANOVA for baited video data, similarity percentages (SIMPER) were used to identify significant distinguishing fish species. This criteria was based on dissimilarity to standard deviation ratios (Diss/SD) >2 and percentage contributions >10%. The mean relative abundance (±1 se) of distinguishing species was then compared between each habitat type. Mean relative abundances were also compared between habitats for the targeted species *Pagrus auratus* and *Sillaginodes punctatus*.

## Results

### Numbers of Fish and Species Recorded by Baited Videos versus Traps

**Figure 3:**
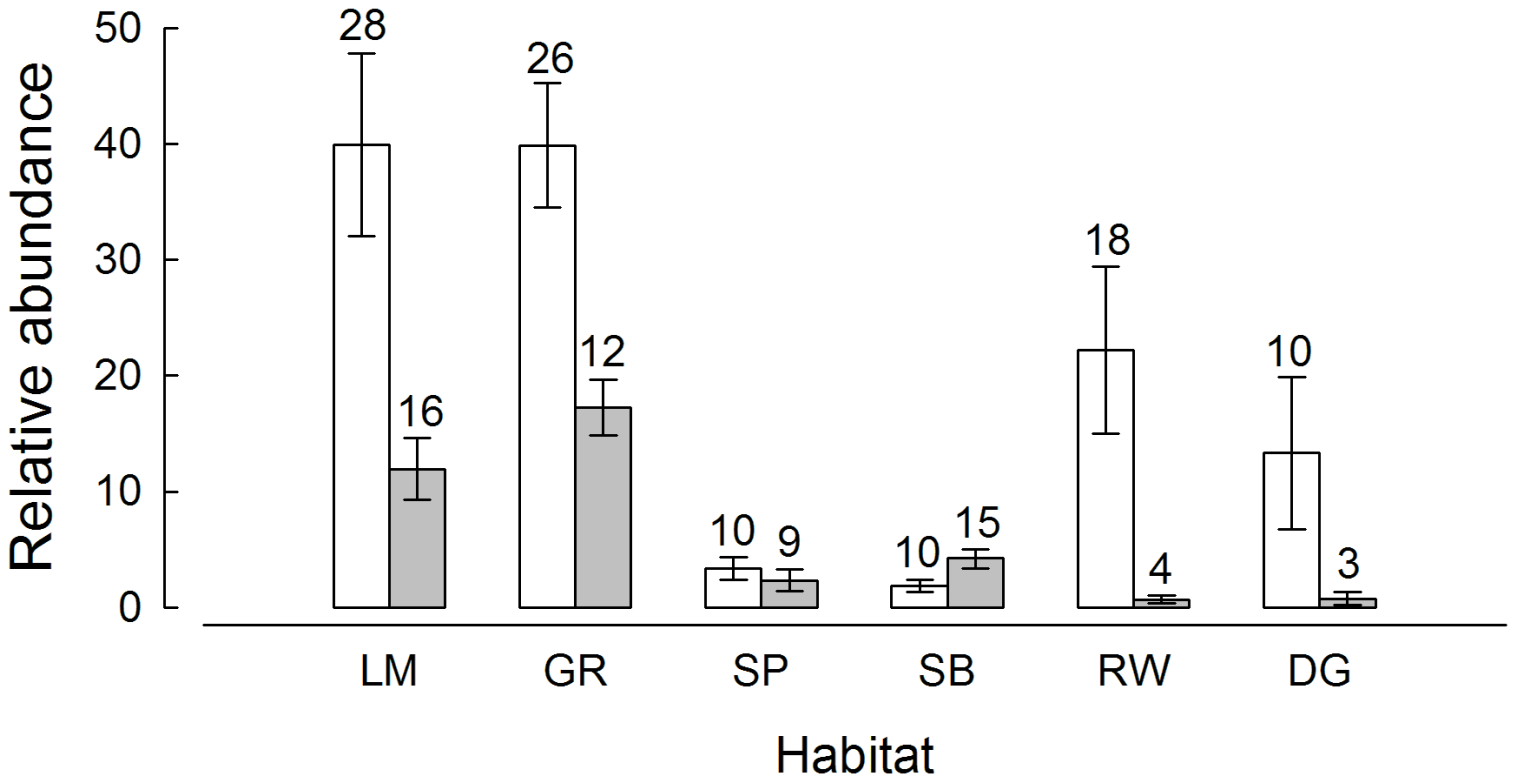
Mean (±1 se) relative abundance (*N_max_*, baited video; numbers, trap) of fish sampled per replicate from each habitat using baited videos (white bars) and traps (grey bars). The numbers of fish species sampled from each habitat by each method are shown above bars. LM, limestone outcrops; GR, seagrass; SP, sand bank (<10 m); SB, sand basin (>10 m); RW, rockwall; DG, dredged channel.

Based on the sum of *N_max_* values, baited videos sampled at least 3,944 individual fish from 43 species and 27 families compared with only 1,040 individuals from 27 species and 18 families in traps. There were only two species sampled by the traps that were not sampled by the baited videos (*Platycephalus longispinis* and *Gymnapistes marmoratus*), whereas there were 16 species sampled by baited videos that were not caught in traps. For all species caught by both methods, a greater number of individuals were recorded from baited videos than in the traps, with the exception of *Pentapodus vitta*. Similarly, for each habitat type the mean abundance and species richness recorded by baited videos were greater than those determined from traps, except at sand basin sites (Fig. 3). This was particularly evident for the modified habitats. In rockwall habitats, baited videos recorded an average of 22.2 fish per replicate (±7.2 se) from a total of 18 species, compared with <1 (±0.3 se) fish per replicate from four species caught by traps (Fig. 3). Likewise, in dredged channel habitats baited videos recorded an average of 13.3 fish per replicate (±6.6 se) from 10 species, compared to traps that captured an average of less than one fish per replicate (±0.6 se) from three species (Fig. 3). The lower mean abundances and species richness recorded by traps may be attributed to the fact that 31% of trap sets caught invertebrate piscivorous predators (predominantly *Portunus pelagicus* or *Octopus* spp.), which were likely to deter fish from entering.

### Fish Assemblage Comparisons between Sampling Methods and Habitats

The fish assemblages differed significantly between sampling methods and among habitat types and an interaction was detected between these two variables (*P* = 0.001, Table 1). Within the natural habitats, both sampling methods recorded higher relative abundances and numbers of species in limestone outcrop and seagrass habitats (∼40 fish per replicate and >25 species) than sand bank and sand basin habitats (<4 fish per replicate and 10 species, Fig. 3). This was reflected in the pairwise comparisons, where based on data collected by baited videos, fish assemblages differed significantly between all natural habitats (*P*<0.025, Table 2), with the two soft sediment habitats, i.e. sand banks and sand basin, only marginally different (*P = *0.041). Similar trends in pairwise comparisons were evident between natural habitats using trap data, except that fish assemblages in sand bank and sand basin habitats were not significantly different (Table 2). Fish assemblages associated with the two modified habitats were poorly sampled using traps compared to baited videos (Fig. 3). At the rockwall locations, baited videos recorded approximately half the relative abundance of fish than in limestone outcrop and seagrass habitats (40 vs 22 fish per replicate, Fig. 3). However, baited videos recorded markedly higher relative abundances of fish from dredged channels than sand bank and sand basin habitats (13 vs 3 fish per replicate, Fig. 3). Pairwise tests comparing fish assemblages of natural and modified habitats with similar physical characteristics, as recorded by baited videos, found no significant differences between dredged channels and both sand banks and sand basin habitats (*P* = 0.273 and 0.180, respectively) and between rockwall and limestone outcrop habitats (*P* = 0.215, Table 2). Fish assemblages recorded by baited videos in seagrass were significantly different from both modified habitats i.e. rockwalls and dredged channels (Table 2). While traps were also unable to detect a significant difference between dredged channel and soft sediment habitats (*P*>0.05), limestone outcrops and rockwall habitats did differ significantly (*P* = 0.026, Table 2).

**Table 1:** PERMANOVA results comparing the composition of fish assemblages between methods and across habitats.

Source	df	SS	MS	Pseudo-F	P
Method	1	18872	18872	13.461	0.001
Habitat	5	55459	11092	7.912	0.001
Method*Habitat	5	17957	3591.3	2.5617	0.001
Residual	90	1.26E+05	1401.9		
Total	101	2.21E+05			

**Table 2:** Results of pairwise PERMANOVA on the composition of fishes recorded with each method between habitats.

Groups			Videos		Traps	
			t	P	t	P
Sand bank	*v.*	Sand basin	1.614	**0.041**	1.505	0.077
Sand bank	*v.*	Seagrass	3.122	**0.001**	2.896	**0.001**
Sand bank	*v.*	Limestone outcrops	2.903	**0.001**	1.966	**0.008**
Sand bank	*v.*	Rockwall	1.806	**0.024**	1.156	0.290
Sand bank	*v.*	Dredged channel	1.282	0.180	0.678	0.694
Sand basin	*v.*	Seagrass	4.068	**0.001**	3.048	**0.001**
Sand basin	*v.*	Limestone outcrops	3.694	**0.001**	1.757	**0.015**
Sand basin	*v.*	Rockwall	2.386	**0.002**	1.666	**0.039**
Sand basin	*v.*	Dredged channel	1.156	0.273	1.253	0.198
Seagrass	*v.*	Limestone outcrops	2.802	**0.001**	2.294	**0.001**
Seagrass	*v.*	Rockwall	1.962	**0.016**	2.206	**0.026**
Seagrass	*v.*	Dredged channel	2.324	**0.006**	2.610	**0.003**
Limestone outcrops	*v.*	Rockwall	1.231	0.215	1.782	**0.026**
Limestone outcrops	*v.*	Dredged channel	1.847	**0.029**	1.888	**0.021**
Rockwall	*v.*	Dredged channel	1.400	0.167	1.336	0.194

Significant differences in bold.

**Figure 4:**
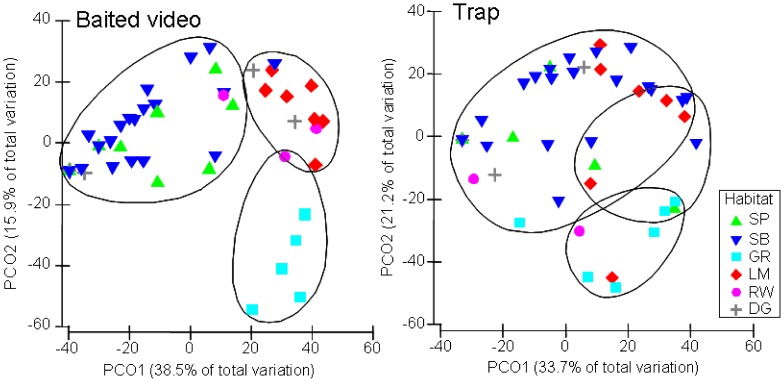
Principal coordinates ordination (PCO) of baited video (left) and trap (right) data overlayed with SIMPROF cluster analysis (35% similarity) defining significant groups. Habitat types include SP, sand bank (<10 m); SB, sand basin (>10 m); GR, seagrass; LM, limestone outcrops; RW, rockwall; DG, dredged channel.

Using an unconstrained PCO ordination and SIMPROF analysis, the natural habitat sites were distinguished into three main groups for both sampling methods at a Spearman correlation >0.35 using Bray-Curtis similarity (Fig. 4). According to the baited video data, these three groupings were clearly discriminated into seagrass, limestone outcrop and soft sediment (combining sand banks and sand basin, Fig. 4). Points for the rockwall habitats were located towards the top right of the plot, with one in each of the limestone outcrop and soft sediment groups and one within an overlap of the limestone outcrop and seagrass groups (Fig. 4). In contrast, the four dredged channel sites were dispersed with two sites in each of the limestone outcrop and soft sediment habitat groups (Fig. 4). According to the trap data, there was greater dispersion of natural and modified habitat sites within the PCO ordination, with many sites included in an overlap between groupings, which was not apparent in the analysis of the composition of fishes recorded by baited video (Fig. 4).

**Figure 5:**
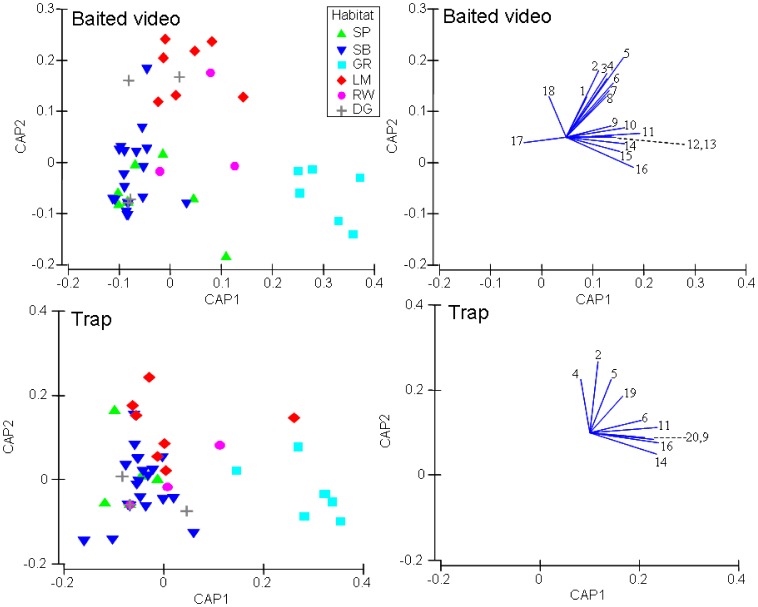
Canonical analysis of principal coordinates (CAP, left) ordination of baited video (above) and trap (below) data with corresponding strength and direction of Spearman correlation >0.35 of fish species shown as line vectors (right). SP, sand bank (<10 m); SB, sand basin (>10 m); GR, seagrass; LM, limestone outcrops; RW, rockwall; DG, dredged channel. Fish species include *Parequula melbournensis* ^1^, *Pseudocaranx sp.* ^2^, *Trachurus novaezelandie* ^3^, *Pagrus auratus* ^4^, *Pentapodus vitta* ^5^, *Notolabrus parilus* ^6^, *Coris auricularis* ^7^, *Upeneichthys vlamingii* ^8^, *Apogon rueppellii* ^9^, *Arripis georgianus* ^10^, *Meuschenia freycineti* ^11^, *Acanthaluteres spilomelanurus* ^12^, *Sillaginodes punctatus* ^13^, *Pelates octolineatus* ^14^, *Sphyraena novaehollandiae* ^15^, *Torquigener pleurogramma* ^16^, *Trygonorhina fasciata* ^17^, *Myliobatis australis* ^18^, *Scobinichthys granulatus* ^19^, *Haletta semifasciata* ^20^.

Plots of the principal coordinates from the constrained CAP analysis showed closer clustering among sites from the same habitats for baited videos than traps (Fig. 5). This was confirmed by the higher values of leave-one-out allocation success and canonical correlation (δ^2^). Fish assemblages associated with natural habitats had an allocation success of 79.5% (δ^2^ = 0.88) when sampled by baited videos, compared to 54.5% (δ^2^ = 0.79) when sampled by traps. For the baited video data, the seagrass and limestone outcrop categories both had 100% allocation success, revealing the lower allocation success was due to misclassification between the two soft sediment groups (i.e. 62.5% for sand banks and 73.9% for sand basin habitats).

### Distributions of Important and Distinguishing Fish Species among Habitats

Using data derived from baited videos, there were distinct groups of fish identified from CAP analysis with relative abundances significantly correlated (Spearman >0.35) with each of the three main natural habitat groups (i.e. seagrass, limestone outcrop and soft sediment, Fig. 5). There were eight fish species with relative abundances significantly correlated with seagrass (*Apogon rueppellii*, *Arripis georgianus*, *Meuschenia freycineti*, *Acanthaluteres spilomelanurus*, *Sillaginodes punctatus*, *Pelates octolineatus*, *Sphyraena novaehollandiae* and *Torquigener pleurogramma*, Fig. 5). Similarly, there were eight fish species with relative abundances significantly correlated with limestone outcrops (*Parequula melbournensis*, *Pseudocaranx sp.*, *Trachurus novaezelandie*, *Pagrus auratus*, *Pentapodus vitta*, *Notolabrus parilus*, *Coris auricularis* and *Upeneichthys vlamingii*, Fig. 5). However, there were only two species of ray, *Trygonorhina fasciata* and *Myliobatis australis*, with relative abundances correlated with the soft sediment group, which most likely reflected the markedly lower abundances and species of fish recorded from these habitats (Fig. 5). In comparison, the CAP analysis of data derived from traps determined that only the relative abundances of a subset of fish species could be associated with seagrass from the other habitats (Fig. 5).

**Figure 6:**
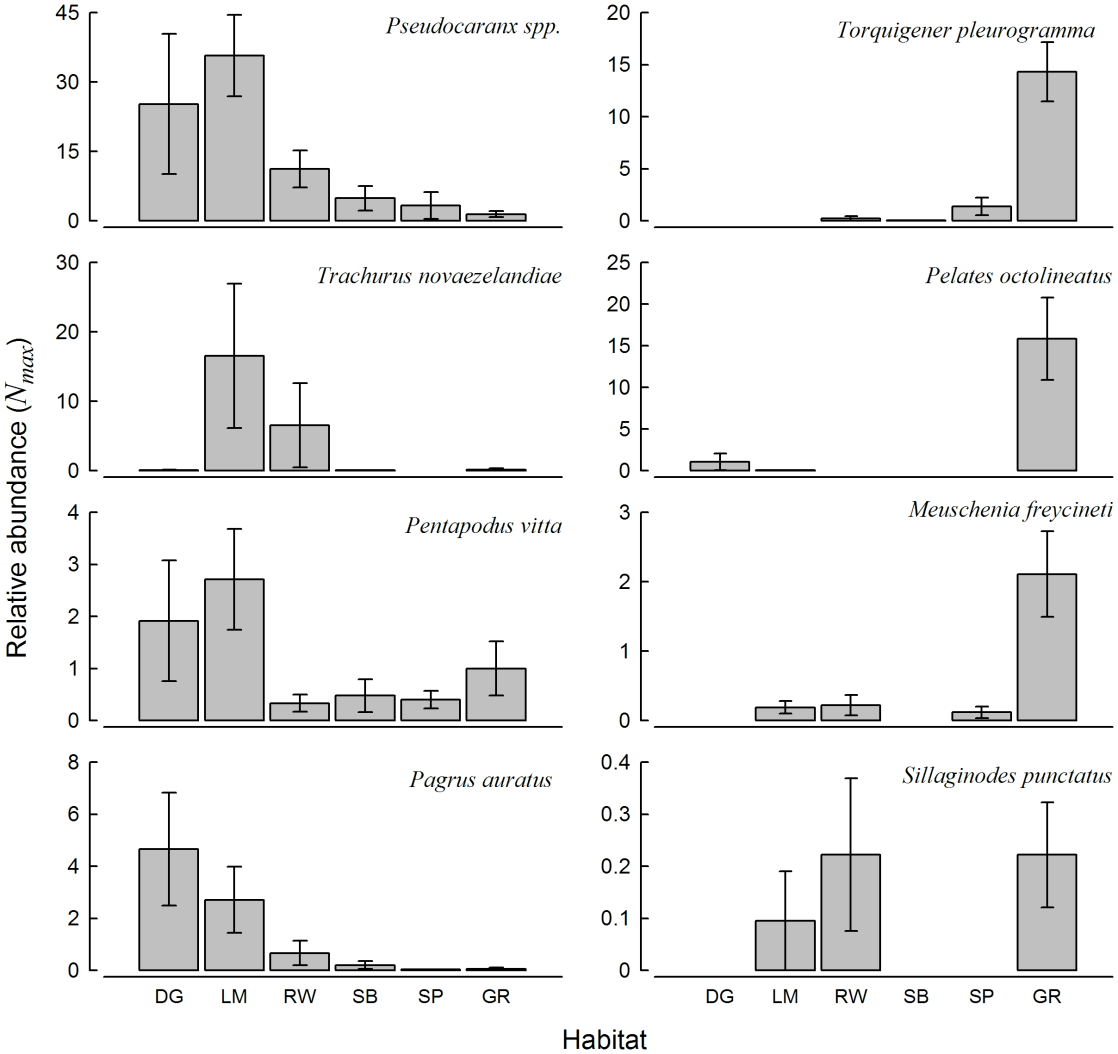
Mean (±1 se) relative abundance per replicate (*N_max_*) of distinguishing and important (commercially and recreationally fished) fish species for each habitat type from baited videos. DG, dredged channel; LM, limestone outcrops; RW, rockwall; SB, sand basin (>10 m); SP, sand bank (<10 m); GR, seagrass.

Based on SIMPER analysis using data derived from baited videos, there were three fish species, i.e. *Torquigener pleurogramma*, *Pelates octolineatus* and *Meuschenia freycineti*, that distinguished fish assemblages associated with seagrass from soft sediment and dredged habitats (contribution >10%, Diss/SD ratios >2.0). Only *T. pleurogramma* distinguished seagrass from limestone outcrop habitats. Notably, each of these three species occurred almost exclusively in seagrass (Fig. 6). There were three fish species including *Pseudocaranx sp.*, *Trachurus novaezelandie* and *Pentapodus vitta* that distinguished assemblages between limestone outcrop and soft sediment habitats. Although the mean relative abundances of *Pseudocaranx sp.* and *P. vitta* were markedly higher in limestone outcrop and dredged channel habitats, they were also recorded consistently but at lower abundances in all other habitats (Fig. 6). In contrast, *T. novaezelandie* was recorded almost exclusively in limestone outcrop and rockwall habitats (Fig. 6).

The distributions of the recreationally and commercially important *P. auratus* and *S. punctatus* were based on 134 and eight observations, respectively, from the 153 baited video replicates. All of the *P. auratus* except one individual were considered to belong to the 0+ age cohort (ca six months of age), based on their length relative to the size of the bait holder and known age-length relationship [10], [43]. The relative abundance of juvenile *P. auratus* was highest in the dredged channel, and to a lesser extent, the limestone outcrop habitats, with occasional occurrences in the remaining habitats (Fig. 6). The ages of the eight *S. punctatus* observed by baited videos were indeterminate based on their relative length, they were however relatively small and most likely from young age classes. The individuals of that species were recorded in three of the six habitat types, including rockwall, seagrass and limestone outcrops (Fig. 6).

## Discussion

Baited videos recorded much greater relative abundances and numbers of fish species than opera-house traps from identical locations in four natural and two anthropogenically-modified habitats. As such, baited videos allowed for greater discrimination of fish assemblages between habitats and were thus considered to be a more efficient sampling method. It is likely that the capture of invertebrate piscivorous predators (i.e. crabs and octopus) in traps (31% of sets) greatly increased the likelihood of predator-prey interactions and thus reduced their sampling effectiveness [44]. Such predator-prey interactions would have been overcome using baited videos, as it is likely the fish would have still been observed within the wide field of view during interactions. Baited video may have also sampled higher numbers of fish species by recording those that were attracted to the bait (evident through their feeding behaviour) as well as those that swam past, thereby increasing the numbers of predatory or scavenging species while also including herbivorous and omnivorous species [45]. The baited video was also able to collect images of the habitat, which facilitated direct links between fish assemblages and their habitat associations that would have not been achieved from the use of traps alone [30]. However, both baited videos and traps were only able to sample relative rather than absolute abundances of fish, given the complexities associated with estimating sampled area based on bait plume dispersal. In comparison, Morrison and Carbines [34] used a towed video method capable of estimating concentrations of juvenile teleosts by calculating swept area.

Fish assemblages sampled in natural habitats by baited videos were discriminated into three distinct groups, i.e. seagrass, limestone outcrops and soft sediment. Within the soft sediment group, there were only marginal differences between fish assemblages in habitats shallower (sand banks) or deeper (sand basin) than ten metres. In comparison, fish assemblages associated with natural habitats, as determined from traps, displayed weak discriminating power and tended to misclassify sites between the three groups, irrespective of whether analysis was constrained by *a priori* habitat classifications.

The majority of habitat in Cockburn Sound is flat and relatively featureless soft sediment, particularly in the deep basin and eastern sand bank areas. Despite occupying the majority of the sound, fish assemblages recorded by baited video in these habitats comprised markedly fewer species (31% of species recorded) in relatively lower abundances. In comparison, the very limited area occupied by seagrass and limestone outcrop habitats had a 15 fold greater abundance of fish and comprised 60% and 65% of all species sampled, respectively. It thus appears important that to maintain fish diversity in Cockburn Sound, the natural seagrass and limestone outcrop habitats need to be conserved, particularly in light of the large scale and continued loss (>77%) of seagrass in this embayment [46].

The anthropogenically-modified habitats were poorly sampled by traps compared to baited videos. This was not surprising for rockwalls, as traps had to be located adjacent to them and relied on fish to leave the high vertical relief and complex shelter provided by the piles of large limestone blocks used in their construction. Baited video could overcome this by being orientated in the direction of the rockwall, thus identifying fish within this complex structure. Discrepancies in efficiency were however surprising for the low relief dredged channels, where the exposure of fish to baited videos and traps would have been similar. These inconsistencies provided little confidence in the ability of traps to provide useful comparisons between natural and modified habitats.

In regards to the overall fish abundance and species richness, the fish assemblages associated with modified habitats did resemble those of natural habitats with similar physical characteristics. Whereby, the overall abundances recorded using baited videos, were not significantly different between rockwall and limestone outcrops and between dredged channel and soft sediment habitats (sand banks and sand basin). Further investigation using PCO and SIMPROF analysis revealed subtle differences in fish assemblages sampled in rockwall sites, where assemblages were found to consist of a subset of species that were recorded from neighbouring natural habitats. This resulted in the three rockwall sites being grouped with one belonging to each of limestone outcrop and sand groups, and one combined within an overlap of limestone outcrop and seagrass groups. This suggests that fish assemblages sampled from rockwall habitat represented an altered composition from that associated with natural limestone outcrop habitat. Thus, the characteristics of rockwall habitat may not have provided the necessary resource requirements (e.g. shelter and/or diet) for some species, resulting in an alteration to the natural trophic structure and interspecific interactions [8], [9].

The fish assemblages sampled by baited video in dredged channel habitat showed mixed results, with two sites included within the soft sediment group, as was hypothesised, and two sites unexpectedly included within the limestone outcrop group. The two dredged channel sites that were grouped within the limestone outcrop group resulted from a high relative abundance of *Pseudocaranx* sp., *P. auratus* and *P. vitta*. The other two dredged channel sites that were included within the soft sediment group consisted of only *T. fasciata* from six replicates. The frequency of dredging of these channels in Cockburn Sound is low (ca every 8–10 years), which suggests a limited number of fish species will recolonise these disturbed areas, but their succession appears variable.

The results of this study support the use of baited videos over traps for broad fish ecology studies [27], and provided a non-extractive application for sampling of predominantly smaller bodied and juvenile fish species in sensitive or turbid environments. It also confirms the advantages of using baited videos for such studies compared to many traditional sampling techniques, such as line, trap and trawl [26], [47], [48], [49]. This method would also be useful for collecting information on the relative abundance of 0+ aged recruits, thus contributing information on recruitment strength toward the stock assessments of exploited teleosts, e.g. *P. auratus* [10]. In addition, this study provides sound quantitative data and repeatable methods for assessing changes in fish communities, which could contribute toward ecological assessments of developments involving anthropogenic modification of marine embayments.
